# *Helicobacter pylori* and T Helper Cells: Mechanisms of Immune Escape and Tolerance

**DOI:** 10.1155/2015/981328

**Published:** 2015-10-07

**Authors:** Tiziana Larussa, Isabella Leone, Evelina Suraci, Maria Imeneo, Francesco Luzza

**Affiliations:** Department of Health Science, University of Catanzaro “Magna Graecia”, 88100 Catanzaro, Italy

## Abstract

*Helicobacter pylori* colonizes the gastric mucosa of at least half of the human population, causing a worldwide infection that appears in early childhood and if not treated, it can persist for life. The presence of symptoms and their severity depend on bacterial components, host susceptibility, and environmental factors, which allow *H. pylori* to switch between commensalism and pathogenicity. *H. pylori*-driven interactions with the host immune system underlie the persistence of the infection in humans, since the bacterium is able to interfere with the activity of innate and adaptive immune cells, reducing the inflammatory response in its favour. Gastritis due to *H. pylori* results from a complex interaction between several T cell subsets. In particular, *H. pylori* is known to induce a T helper (Th)1/Th17 cell response-driven gastritis, whose impaired modulation caused by the bacterium is thought to sustain the ongoing inflammatory condition and the unsuccessful clearing of the infection. In this review we discuss the current findings underlying the mechanisms implemented by *H. pylori* to alter the T helper lymphocyte proliferation, thus facilitating the development of chronic infections and allowing the survival of the bacterium in the human host.

## 1. Introduction

*Helicobacter pylori* is a human pathogen responsible for an infection involving nearly half of the world's population, frequently associated with chronic inflammation of the gastric mucosa that can lead to peptic ulceration and gastric cancer in particularly susceptible individuals [1, 2]. Infection is commonly acquired during childhood and, if not treated, the host can carry the bacterium even for life, mounting an innate and adaptive immune response which is unable to clear the pathogen [3]. Indeed the hallmark of* H. pylori* is its ability to escape host defence mechanism with several not yet entirely clarified strategies involving both the innate and adaptive immune systems of the host [4]. Many studies demonstrated that specific T helper (Th) cell subsets and their signature cytokines contribute to the control of the infection and sustain the development of the chronic inflammation. Most data support the critical role of these interactions in the pathogenesis of* H. pylori*-related diseases such as adenocarcinoma [5]. In this review, we discuss how* H. pylori* manipulates the responses of the T helper cells, avoiding its clearance by the host immune system.

## 2. The Interplay between* H. pylori* and the Effective T Helper Lymphocytes

### 2.1. T Helper-Mediated Cell Immunity in Chronic* H. pylori*-Induced Inflammation

Naïve T CD4+ helper (T CD4+) cells can be induced to differentiate towards T helper 1 (Th1), Th2, Th17, and regulatory (Treg) phenotypes according to the local cytokine milieu. T helper cells were historically divided into the two functional subsets, Th1 and Th2, characterized by distinct patterns of cytokine secretion. Th1 cells produce interleukin- (IL-) 2 and interferon- (IFN-) *γ* and promote cell-mediated immune responses, whereas Th2 cells secrete IL-4, IL-5, IL-6, and IL-10 and induce B cell activation and differentiation. In general, most intracellular bacteria induce Th1 responses, whereas extracellular pathogens stimulate Th2-type responses. Recently, the Th1/Th2 cell paradigm was enriched with another subset of T helper cells, called Th17, since they were identified as the source of IL-17. These cells are characterized as producers of IL-17A, IL-17F, IL-21, and IL-22 and are involved in host defensive mechanisms to various infections, especially extracellular bacterial infections, but also in the pathogenesis of autoimmune diseases [6]. Regulatory T cells (Treg) are naturally occurring T cells which are capable of suppressing effector T cell proliferation and cytokine production. Thereby they play a critical role in maintaining peripheral tolerance, moderate the immune response to pathogens by regulating the balance between immunity and inflammation, and prevent severe multiorgan autoimmune diseases [7]. The condition of chronic antral gastritis following* H. pylori* infection is characterized by a cellular inflammatory infiltrate which displays feature of both innate and adaptive immune response. Of the latter, the T CD4+ cells are considered the main actors in the establishment of chronic inflammation [8]. The adaptive immune response mounted by the host against* H. pylori* has been shown to include both Th1 and Th17 components, which are implicated in infection control through multiple pathways, as well as the Th2-derived cytokines, that have been detected in* H. pylori* infection although their role is not well understood [9].

#### 2.1.1. Th1 Cells

Although the acquired immune response to* H*.* pylori* is composed of both Th1- and Th2-type cells, cytokine profiles indicate predominance of a Th1 response. Th1 are involved in immune response to many pathogens mostly by providing a source of IFN-*γ*. Indeed, as naïve T cells differentiate into Th1 cells, they will produce IFN-*γ* whose increased levels establish a Th1 dominant microenvironment and at the same time inhibit IL-2 production, which is necessary for Th2 response [10]. The Th1 proliferation in gastric mucosa infected by* H*.* pylori* involves signals provided by antigen-presenting cells and cytokines produced in response to the components of the pathogen, such as LPS, resulting in enhanced secretion of IFN-*γ* itself, IL-12, and IL-18 [11]. T-bet (T-box expressed in T cells) is a transcription factor that is required for differentiation of T CD4+ cells and their secretion of IFN-*γ* and hence holds a central role in the development of gastritis due to* H. pylori*. Mouse models have shown that T CD4+ cells from mice lacking T-bet fail to express IFN-*γ* and thus are limited to non-Th1-type responses [12].

#### 2.1.2. Th2 Cells

Several reports indicated a role for Th2 phenotype in protection from infection. When a Th2 cell line from mice immunized/challenged with* Helicobacter felis* was transferred adoptively in naïve recipients before live bacterial challenge, they showed a dramatic reduction in bacterial load. On the other hand, increased numbers of bacteria were noted in IL-4-deficient mice [13]. Therapeutic mucosal immunization of mice with a recombinant* H. pylori* urease B subunit had been proven to induce progressively a Th2 cell response resulting in the elimination of the pathogen. Nevertheless, the mice did develop robust histologic gastritis upon challenge, consistent with a Th1-driven proinflammatory response, thus lessening the supposed role of Th2 pathway in preventing the* H. pylori* related diseases [14]. Consistent with these findings, Garhart et al. demonstrated that vaccine-induced protection is obtained in IL-4 deficient mice, suggesting that a Th2 response is not necessary for protection, although it could still play a role in the situation that some clearance mechanisms become redundant [15].

#### 2.1.3. Th17 Cells

Many reports support the involvement of Th17 cells in the inflammation sustained by* H. pylori* in humans. Gray et al. showed that transfer of CD4+ T cells from mice that are deficient in IFN-*γ* or T-bet does induce gastritis, even if in a lower grade compared with C57BL/6 mice [16]. Thus, while IFN-*γ* clearly contributes to gastritis, Th1 cells do not appear to be essential, supporting the proinflammatory role of the IL-17. Also in human infection an enhanced production of IL-17 was found to perform a regulatory activity on the strong neutrophil chemoattractive cytokine IL-8, which plays a major role in the* Hp*-associated acute inflammatory response, as demonstrated by the significant inhibition of IL-8 production after neutralization of IL-17 [17].

#### 2.1.4. Treg Cells

Since T effector cells are limited in their proliferation and function by Treg cells, elevated levels of Treg cells were verified in infected human gastric mucosa and also* H. pylori*-specific Tregs have been found in the circulation of infected individuals [18]. This evidence suggests that* H. pylori* colonization results in expansion of the Treg population and their recruitment to the site of infection limits the inflammatory response, thus representing a mechanism of pathogen persistence. However, reports investigating the role of Treg cells in* H. pylori* infection are controversial and far from a conclusion. Several studies suggest that the local Treg response protects the gastric mucosa from exaggerated inflammation and tissue damage, and the risk of* H. pylori*-related diseases is inversely related to Treg accumulation, even if the reduction of the inflammatory response achieved by Treg leads to increased bacterial density. The inability to mount a protective inflammatory response is responsible for the establishment of a chronic infection and in some patients for the development of atrophic gastritis and gastric cancer progression [19]. Other reports suggested a role for Tregs in modulating tumour growth. Indeed, Treg cells might be important in the cross-talk between neoplastic MALT B-cells and T-cells specifically activated by* H. pylori* antigens since the number of Treg cells had a positive influence in the response to antibiotic therapy [20]. These findings raised the possibility of a suppressive influence of these Tregs on the T-cell population responsible for tumour growth maintenance through the presentation of* H. pylori* antigens. Treg cells have been shown to downregulate the Th1 response toward* Bordetella pertussis* and* Leishmania major*, leading to prevention or retardation of pathogen eradication [21, 22]. In the context of* H. pylori* infection, Treg depletion led to an enhanced T CD4+ cell activation, which is responsible for the reduction in bacterial load. Consistently,* H. pylori*-infected mice lacking in Treg cells develop a severe gastritis, but not athymic mice, accounting for the suppressive role of Treg cells on Th1 response [23]. On the other hand, Treg depletion in BALB/c mice resulted in an increased production of Th2 cytokines by lymphocytes in response to* H. pylori* antigens, suggesting a skewing towards a Th2 response [24].

### 2.2. The Impaired Th1/Th2 Response as a Way of Escape for* H. pylori*

Studies based on animal models confirmed the predominant Th1 phenotype, since infected or immunized/challenged mice demonstrated local and systemic production of IFN-*γ* and undetectable levels of IL-4 or IL-5. Cellular proliferation correlated with the severity of the gastritis score and* in vivo* neutralization of IFN-*γ* resulted in a significant reduction of gastric inflammation [25]. In humans, Th responses to* H. pylori* have been known for a long time to be strongly biased toward Th1. The pattern of cytokines produced by the immunologically active cells in the gastric antrum of infected individuals was analyzed and revealed the prevalence of IFN-*γ*, TNF-alpha, and IL-12, providing evidence for Hp-specific Th1 effector cells [26]. Despite these data, a growing number of reports, such as the one of Eaton et al. carried out in mice, suggest that this Th1-biased response is dysfunctional and may play an important role in pathogenesis of the* H. pylori*-related diseases [27]. The reasons for the impaired Th1 immune response could lie in the continuous process of virulence factor elaboration implemented by the bacterium over the thousands of years of coexistence with the human host.

#### 2.2.1. The Vacuolating Cytotoxin Inhibits T CD4+ Cells Activation

The vacuolating cytotoxin (VacA), initially identified due to its ability to induce vacuolization of epithelial cells, has also been revealed as an inhibitor of T cells signaling and proliferation by inducing a G1/S cell cycle arrest through the interference with the T cell receptor/IL-2 signaling pathway at the level of the Ca2+-calmodulin-dependent phosphatase calcineurin. In this way, VacA avoids the nuclear translocation of nuclear factor of activated T cells (NFAT), the main regulator of the T cell pathway, resulting in the downregulation of IL-2 gene transcription [28]. Further experiments indicated that VacA suppresses IL-2-induced cell-cycle progression and proliferation of primary human T cells without affecting IL-2-dependent survival, but through its N-terminal hydrophobic region necessary for the formation of anion-selective membrane channels causing the arrest of the clonal expansion of T cells already activated by* H*.* pylori* antigens [29].

#### 2.2.2. *H. pylori* Gamma-Glutamyl Transpeptidase and Arginase Impair T Cells Proliferation

A low-molecular-weight protein of* H. pylori* has been reported to inhibit proliferation of T cell lymphocytes by blocking cell cycle progression at the G1 phase through G1 cyclin-dependent kinase activity modulation [30]. Using functional experiments, Schmees et al. identified this suppression factor of T cells as the gamma-glutamyl transpeptidase (GGT) secreted by* H. pylori*. Since this enzyme mediates the extracellular cleavage of glutathione, with ROS production and consequently induction of cell cycle arrest in lymphocytes, the authors demonstrated that recombinantly expressed GGT showed antiproliferative activity while mutagenesis of GGT in different* H. pylori* strains completely abrogated this inhibitory effect [31]. An additional effective strategy of immune evasion implemented by the bacterium is attributable to* H. pylori* arginase, which is important for urea production by hydrolyzing L-arginine to urea and ornithine. Knowing that L-arginine is required for T cell activation and function, Zabaleta et al. incubated* H. pylori* wild type and arginase mutant bacteria with T cells and revealed that arginase caused a significant decrease in T cell proliferation by depleting L-arginine availability, but not in coculture with the arginase mutant strain. In addition, arginase inhibitors reversed these events. The results did not appear to be mediated by apoptosis because less than 10% of cells became annexin V positive in all experiments, but rather were correlated with a reduced expression of the chief signal transduction protein CD3*ζ*-chain of the T cell receptor (TCR), which is required for the initiation of T cell activation [32].

#### 2.2.3. The Cytotoxin-Associated Gene Pathogenicity Island Induces T Cell Death

Another virulence factor of* H. pylori* involved in T cell function impairment is the cytotoxin-associated gene pathogenicity island (*cag* PAI), associated with a more aggressive phenotype of disease. Although the stimulation with viable strains of* H. pylori* with or without the* cag* PAI induced apoptosis in epithelial cells, T cell death was only observed using the* cag* PAI-bearing strains of the bacteria, through the induction of Fas ligand (FasL). This mechanism was found to be able to limit host immunity through the induction of T cell death in a Fas-dependent manner whereas inhibiting protein synthesis blocked FasL expression and apoptosis of T cells [33].

#### 2.2.4. Modulation of Th1/Th2 Pathway by Cyclooxygenase and Indoleamine 2,3 Dioxygenase

Cyclooxygenase (COX) is an enzyme that catalyzes the conversion of arachidonic acid into prostaglandins. It exists in two isoforms, the constitutive isoform COX-1 and the inducible isoform COX-2, the latter being involved in the inflammatory response. Indeed, COX-2 activation suppressed Th1 polarization in response to* H. pylori* preparations in human peripheral blood mononuclear cells and has been shown to be upregulated in* H. pylori*-colonized gastric mucosa [34]. With this in mind, we provided evidence that an enhanced expression of COX-2 occurs during* H. pylori* colonization of the human stomach and may induce downregulation of Th1 signaling pathway, thus representing a mechanism by which* H. pylori* may actually interfere with normal T-cell activation in human gastric mucosa [35]. In a further study, we expanded this hypothesis showing that* H. pylori*-induced enhanced expression of indoleamine 2,3 dioxygenase (IDO) may modify the Th1/Th2 balance [36]. IDO is a heme-containing enzyme that catalyzes the first and rate-limiting step in tryptophan degradation via the kynurenine pathway. The consequent tryptophan starvation in the microenvironment limits T cell replication and induces T-cell apoptosis, hence impairing the Th1 response. Using functional experiments in* ex vivo* obtained gastric biopsies we demonstrated that the expression of IDO was higher in* H. pylori*-infected samples compared with uninfected samples and that its inhibition leads to increased levels of IFN-*γ* and T-bet while IL-4 production was reduced.

#### 2.2.5. Stromal Factors Impair Th1 Response

Together with bacterial factors, local host factors can contribute to the permissive mucosal environment in* H. pylori* gastritis. Epithelial cells, resident immune cells, and lamina propria stromal cells secrete a series of cytokines, chemokines, and other soluble factors in the gastrointestinal mucosa, which are stored in the extracellular matrix, as a reservoir of immunoactive “stromal factors.” Mucosal dendritic cells (DCs) are of particular importance in initiating the Th1 response to the bacterium, by releasing of IL-12. DCs were found to be suppressed in their activation against* H. pylori* by the presence of stromal factors in gastric and intestinal mucosa capable of downregulating DC responsiveness to* H. pylori* and thus resulting in a dampened gastric Th1 response [37].* H. pylori* was found to induce tolerogenic DCs, unsuitable to elicit an effective and strong T cell recruitment also via the novel receptor for* H. pylori* on DCs called dendritic cell-specific ICAM-3-grabbing nonintegrin (DC-SIGN). Indeed, Lewis antigen expression by* H*.* pylori* LPS was shown to bind to DC-SIGN C-type lectin present on gastric DCs and this interaction blocked Th1 cell recruitment, whereas Lewis-negative* H. pylori* strains enhanced Th1 cell development [38].

Prostaglandin (PGE) 2 is another important stroma-derived mediator, which, in addition to the inhibitory effect on* H. pylori*-induced DCs activation, has the capacity to downmodulate directly T lymphocytes. Indeed, in a mouse model, Th1 cells fail to migrate, proliferate, and secrete cytokines when exposed to PGE2* in vitro* and* in vivo*, as the result of the silencing of interleukin-2 gene transcription [39].

**Figure 1:**
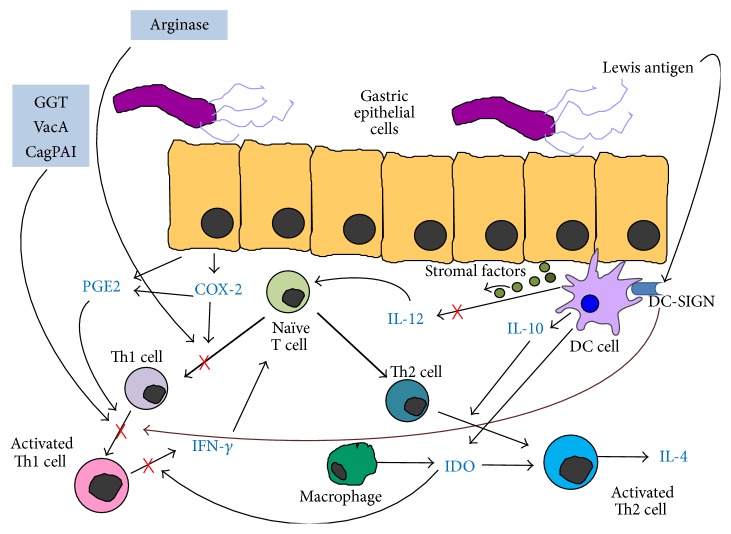
The biased Th1/Th2 cells response. The vacuolating cytotoxin (VacA) and the gamma-glutamyl transpeptidase (GGT) secreted by* H. pylori* inhibit Th1 cell proliferation by inducing a G1/S cell cycle arrest [28–31], while the cytotoxin-associated gene pathogenicity island (*cag* PAI) promotes Th1 death by the induction of Fas ligand [33]. The committed T naïve cells are unable to differentiate into Th1 line due to the enzyme arginase possessed by* H. pylori* [32].* H. pylori*-induced cyclooxygenase- (COX-) 2 activation, alone and in conjunction with its product prostaglandin (PGE), suppresses Th1 polarization [34, 35, 39]. The* H. pylori*-induced expression of indoleamine 2,3 dioxygenase (IDO) limits the IFN-*γ* production by Th1 cells and favours the activation of Th2 cells [36]. Stromal factors suppress the IL-12 production by mucosal dentritic cells (DCs) [37], whose dendritic cell-specific ICAM-3-grabbing nonintegrin (DC-SIGN) receptor interacts with Lewis antigen expressed by* H. pylori* thus blocking Th1 cell recruitment [38].

The above-mentioned mechanisms, as a possible means of* H. pylori* evasion from the effective T helper lymphocytes, are summarized in Figure 1.

### 2.3. Does the Balance between Th17 and Treg Play a Key Role in the Tolerance?

Recently it was assessed that* H. pylori* induces Th17 cell differentiation and impairment of this process could be involved in the shifting of T-cell responses which favours the persistence of the infection. IL-17 is the signature cytokine produced by Th17 cells and has a mediator role in the host inflammatory defence against bacterial and fungal pathogens, particularly at mucosal surfaces [40]. Although increased IL-17 expression is observed during chronic gastric inflammation, the levels produced are not sufficient to clear the infection. Accordingly, in* ex vivo* experiments on human gastric biopsy specimens, it was shown that IDO, which is highly expressed in* H. pylori*-infected gastric mucosa, downregulates IL-17 [36].

Commitment of Treg cells during* H*.* pylori* infection was demonstrated to be affected by B7 family ligands and their receptors, which are expressed on human epithelial cells and play important roles in the growth, development, and differentiation of T cells. At the same time, gastric epithelial cells (GECs) express enhanced levels of B7-H1 and this could contribute to the suppression of CD4+ effector T cell activity and upregulation of Treg cells [41].

On the other hand, B7 family ligands are involved in Th17 response. Indeed, a subsequent study investigated the impact of* H. pylori* and its major virulence factor CagA on the modulation of B7-H2 in gastric mucosa. Using* in vitro* and* in vivo* studies, the authors showed that the downregulation of B7-H2 on GECs was operated by* H. pylori* through the presence of CagA cytotoxin, and that this fact correlated with the decrease in Th17 responses and the enhanced level of* H. pylori* colonization in mice [42].

Kao et al. suggest that a suboptimal Th17 response could lead to the failure of eradication and this would happen because* H. pylori* alters the DC-polarized Th17/Treg balance toward a Treg-biased response, thus suppressing the effective* H. pylori*-specific Th17 immunity. Using animal models, the authors showed that bone marrow-derived DCs pulsed with* H. pylori* skewed the response toward Treg differentiation by a VacA/CagA-independent, transforming growth factor- (TGF-) *β*/IL-10-dependent mechanism, rather than induction of a strong Th17 activation. Accordingly, the production of cytokines such as IL-17, IL-6, and IL-23 was found to be reduced. Moreover, functional experiments of Treg depletion showed the enhancement of the* H. pylori*-specific Th17 response. This correlated with decreased bacterial density and validated the major role of Th17 immunity in bacterial clearance [43]. Another report investigated the tolerogenic properties of* H*.* pylori* on DCs through the involvement of IL-18, a cytokine which acts directly on T cells and promotes their conversion to Tregs. The authors showed that the secretion of IL-18 is induced in DCs upon infection with* H. pylori* and suggest a key role for DC-derived IL-18 in skewing T cell differentiation away from Th17 and toward Treg responses [44]. The tolerogenic activity of DCs depends on inflammasome activation, which allows the release of interleukin-18 from preformed granules. Of note is the role of* H. pylori* in promoting the proteolytic processing of IL-1*β* and IL-18 induced by the inflammasome and caspase-1. This confirms the active presence of the bacterium in restricting pathogenic Th17 responses and favouring T-regulatory functions, supporting a pivotal role in the commitment of Treg cells [45]. New insights into the mechanisms underlying the development of* H. pylori-*associated complications derive from other experiments on Treg cells. Evidence that pathogens could take advantage of the suppressive function of Tregs on T effector cells has been shown previously in the context of* Leishmania major* and* Helicobacter hepaticus* infections, both conditions in which an increased number of Treg cells prevent the clearance of infection and limit the inflammatory response [22, 46]. However, the presence of chronic inflammation despite the existence of elevated numbers of Tregs suggests that these Tregs have impaired ability to suppress local inflammation.

Based on reports of elevated Treg numbers in* H. pylori* infected sites, a recent study investigated the direct and indirect effect of* H. pylori* on Treg proliferation and function* in vitro* as well as in gastric tissue biopsies from subjects infected with* H. pylori*. The hypothesis was that the bacterium is able to instruct DCs to stimulate proliferation of Tregs and that this happens together with a modulation acted by local factors which impairs the efficiency of Treg cells themselves. As expected,* H. pylori*-stimulated DCs drive Treg proliferation, but their suppressive function was expressed to a less extent and this was due to the production of IL-1*β* enhanced by the bacterium [47]. These data could conclude that Treg expansion in response to* H. pylori*-driven DCs is short-lived and the efficiency of Treg-mediated suppression might be expected to decline after the initial peak. Addressing the central role of IL-1*β* in mediating the effects of* H. pylori* on Tregs is of particular interest, because virulent strains of* H. pylori* expressing cagPAI are associated with elevated levels of IL-1*β*. In this context, further studies on polymorphisms in IL-1*β* could better define the mechanisms of immune evasion and interactions between* H. pylori* and the host.

**Figure 2:**
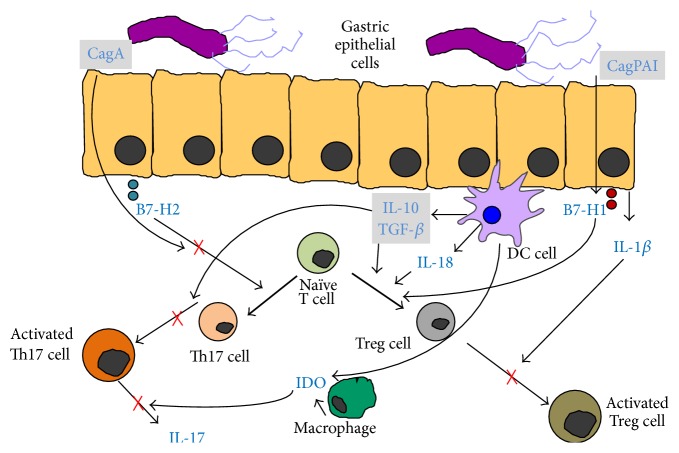
The impaired Th17/Treg balance. The induction of Treg cell differentiation is mediated by the overexpression of B7-H1 due to* H. pylori cag* PAI [41]. On the other hand, the CagA-induced lowered B7-H2 signaling inhibits Th17 cells development from naïve T CD4+ cells [42]. The higher expression of IDO downregulates IL-17 production [36]. DCs promote Tregs differentiation through higher production of IL-18 [44] as well as by a transforming growth factor- (TGF-) *β*/IL-10-dependent mechanism, which in turn blocks the Th17 cell proliferation [43]. The efficiency of Treg cells themselves is impaired by the production of IL-1*β* enhanced by the bacterium [47].

The events described above could explain the state of tolerance implemented by the bacterium and are summarized in Figure 2.

### 2.4. The Immunological Context of Early and Late* H. pylori* Infection

Since most of infected persons acquire the bacterium during early childhood, the study of* H. pylori* infection in children offers the opportunity to investigate early mucosal responses to the bacterium in the human host. The majority of children are infected at a very young age and the risk of infection declines rapidly after 5 years of age. Age, a low socioeconomic status, limited living space, sharing of beds, a low parent education level, pollution of daily used water, and* H. pylori* infection in family members (especially the mother) are the known risk factors for infection [48]. Little is currently known about the immune response to the bacterium during early childhood, especially on factors promoting the spontaneous clearance of the infection that seems to be particularly high in this setting [49]. As in adults, gastric mucosal inflammation always characterizes* H. pylori* colonization in children although the degree of gastric inflammation is significantly less compared with that of adult subjects, in spite of the same* H. pylori* genotype and similar levels of colonization and CagA and VacA status [50]. Only a few studies evaluated the local cytokine profile in children. Results are somewhat conflicting, but they most consistently showed that* H. pylori* infection in children induces the production of proinflammatory cytokines according to a Th1 profile, similar to studies in adults, together with a higher IL-17 expression which correlated with bacterial density [51]. On the other hand, differences in mucosal immunopathology of infected children have been suggested. Lopes et al. found that local cytokine expression appeared to be smaller in* H. pylori*-infected children than in adults and did not correlate with antrum inflammation scores. Moreover, levels of IFN-*γ* were not so enhanced and moderate levels of IL-4, the main Th2 cytokine, were also found, in contrast to data from adult populations [52]. The fact that there was no clear Th1 dominance may indicate that children are more prone to mounting a gastric Th0 or Th2 response than adults or it may be due to a reduced capacity of T CD4+ cells from children to produce IFN-*γ* compared to adult T cells [53]. A study involving 245 children from Latin America showed a lower gastric concentration of IL-2 and IFN-*γ* in the infected children than in the infected adults, supporting the fact that Th1 immune response to* H. pylori* infection varies according to the age of the patients [54]. A reduced neutrophil accumulation in infected children gastric mucosa was found together with significantly lower levels of gastric Th17 cells and IL-17-specific mRNA and protein compared to infected adults [50]. Since IL-17 participates in the recruitment and activation of polymorphonuclear cells that are considered relevant to the clearance of the* H. pylori*, these findings may explain the lower degree of mononuclear and polymorphonuclear cell gastric infiltration observed in infected children than in adults, which is not attributable to differences in the gastric bacterium density. Nevertheless, further data has come from a parallel study recruiting infants, children, and adults. Indeed, PBMCs from infants showed the highest levels of production of IL-17 whereas cells from children produced slightly less and the lowest amounts were produced by adult cells from* H. pylori*-infected subjects. This could be due to the increased production of IL-1*β* by monocytes from infants, which is a strong activator of Th17 response [55]. A recent work compared the frequency of gastroduodenal ulcers in infected children and adults and investigated the effect of chronological age on severity of gastritis and also on NF-*κ*B activation, a transcriptional factor for inflammatory genes induced by* H. pylori*. A positive correlation was found between age and densities of neutrophils and CD3, but not of CD8 or CD20 cells, while NF-*κ*B-p65-positive cells were increased only in infected adults as well as NF-*κ*B-binding activity. Moreover, peptic ulcer disease was less frequent in children than in adult infected subjects, maybe due to the lower mucosal immune response displayed in children [56]. Further intriguing data indicate that Tregs have an important role in regulating the early gastric mucosal inflammatory response to* H. pylori*. It seems that the gastric immune response is not only downregulated in children with* H. pylori* infection but also directed toward a sort of tolerance, which is relevant to the outcome of infection. Studies in animal models demonstrated that neonatally infected mice fail in the local and systemic responses to the bacterium but not adult-infected mice, providing evidence for a state of tolerance due to the induction of peripheral tolerogenic Treg, which efficiently control effector T-cell responses against* H. pylori* [57]. A Korean study demonstrated that the number of FOXP3-expressing Treg cells and the grade of TGF-*β*1 expression were significantly increased in* H. pylori*-positive children compared to the negative group and correlated positively with* H. pylori* density [58]. In children, the degree of generation of* H. pylori*-specific Treg cells seems to depend largely on the age at the time of infection, since* H. pylori* infected children have increased levels of FoxP3-expressing Treg cells and reduced gastric pathology compared with adults. Concurrent with the reduced gastric inflammation and the high number of Treg cells, the levels of Treg cytokines such as TGF-*β* and IL-10 were strongly increased in the gastric mucosa of* H. pylori*-infected children suggesting a pivotal role of Treg participation in the reduced Th1-mediated gastritis and ulceration in these subjects [59]. IL-23 participates in the expansion and maintenance of the Th17 lymphocytes. Children with* H. pylori* infection displayed lower gastric levels of IL-23 compared with adults, and this could prevent the amplification of the shifted Th17 cells, thus resulting in the predominance of Treg instead of Th17 cell differentiation and suggesting that the education of gastric Tregs to establish tolerance to the bacterium begins during early childhood infection [60]. Children and adults immunological features are summarized in Table 1.

## 3. Conclusions

*H. pylori* has coexisted with its human host for at least 30.000 years undergoing an evolutionary adaptation. In contrast to the majority of bacterial pathogens, which temporarily cause virulent disease and then are cleared by the pathogen-specific adaptive immune response,* H. pylori* successfully establishes a persistent infection in its host in spite of the presence of vigorous innate and adaptive immune response. The colonization can persist for decades or for life.* H. pylori* elaborated several evolutionary adaptations that allow the bacterium not only to escape detection by pattern recognition receptors on innate immune cells, but also to evade adaptive immunity. The host mediated immune response fails to clear* H. pylori* and also favours its colonization; hence bacterial virulence factors together with host factors determine the severity of disease. The role of T CD4+ cells is crucial in the immune response to the bacterium, with a Th1 polarized proliferation that was characterized first. Recently, studies showed that the impairment of this Th1 response may be implicated in maintaining the infection, along with the emerging role of other T CD4+ cell subsets, including Treg and Th17 cells. Although multiple studies investigated how all these events are possible, the full scenario is still unclear. Furthermore, little is currently known about the role of T cell subsets in controlling* H. pylori* infection in children, which with its intriguing mechanisms could provide a useful model into the early host response to the bacterium.

The exact role of these subsets of T helper cells in* H. pylori* infection is far from being fully understood. Indeed, the immunomodulatory properties of the pathogen reprogram the immune system towards immunological tolerance and* H. pylori* escape. Even if the Th1 and Th17 panel seems to be involved in the proinflammatory activity of the bacterium, neither Th1 nor Th17 cells are by themselves capable of a spontaneous clearance of the infection. Realistically, a more pronounced inflammation during the early phase of infection could switch the events towards eradication. The increase in Treg cells and the parallel downregulation of Th1 response observed in children compared with infants suggest that the conflict between persistent infection and clearance is decided in the early phase of infection. In this context, Th2 response has been implicated in reducing bacterial load but its protective role is still controversial. On the other hand, the benefit in limiting local inflammation and tissue damage derives from the Treg pathway, but at the same time this leads to an increase in bacterial density and a persistent infection.

Studies on the relationship between* H. pylori* density and acute and chronic inflammation showed intriguing results but it is controversial whether the hallmark of protection is the degree of inflammation or the bacterial load. Indeed,* in vivo* depletion of Treg cells in infected mice was associated with an increased gastric inflammation and reduced bacterial colonization [61]. Enhanced inflammation in IL-10-knockout mice or in immunized mice leads to dramatic reduction of colonization and even clearance of* Helicobacter* species from the stomach [62]. However, a high grade of inflammation often progresses to gastric atrophy with disappearance of* H. pylori* from the stomach and development of an atrophic gastritis [63]. With regard to vaccination, the aim is to eliminate or reduce bacterial load when immunizing therapeutically and several reports showed that the immune responses are activated by the vaccination strategy, but there are few instances of achieving a reduced bacterial load [64]. Detailed studies on the protective T helper cell response have been published and have provided evidence that protection was dependent on Th1 responses. It is now generally accepted that the induction of increased inflammation by Th1 or Th17 cells is the starting point for a protective immunity [65]. Despite previous reports suggesting a protective role for Th2 cells [13, 14], more recent studies did not confirm this hypothesis [15, 66]. An intriguing argument is that protection could follow after an acute contact with* H. pylori*, sustained by an inflammation strong enough to clear the infection.

**Figure 3:**
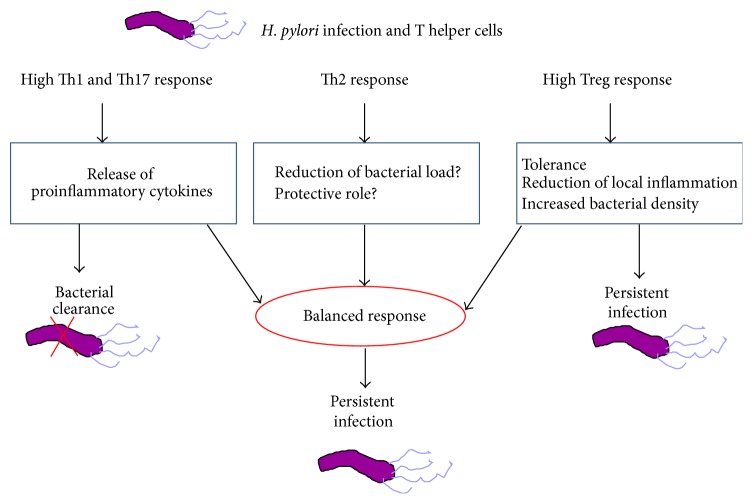
The interplay between* H. pylori* and the effective T helper lymphocytes. Although Th1 and Th17 pathways are both responsible for promoting inflammatory activities during* H. pylori* infection, neither Th1 nor Th17 cells are by themselves capable of a spontaneous clearance of the infection. It could be due to an impaired release of cytokines, suggesting that a more pronounced inflammation during the early phase of infection could switch the events towards eradication. Th2 response has been implicated in reducing bacterial load but its protective role is still controversial and deserves further investigation. Treg cells limit local inflammation and tissue damage but at the same time this fact favours a tolerogenic status which leads to a persistent infection. This complex interplay suggests that the conflict between persistent infection and clearance is decided in the early phase of infection.

The events described above are summarized in Figure 3.

## Figures and Tables

**Table 1:** Children and adults *H. pylori*-related immunopathological features.

Feature	Children versus adults	References
Colonization level	=	[50]
Virulence factors (CagA/VacA)	=	[50]
Bacteria genotype	=	[50]
Polymorphonuclear and mononuclear cell infiltration	↓	[50]
Th2 response	↑	[52]
Th1 response	↓	[53]
T reg response	↑	[59, 60]
Th17 response	↓	[50]
Duodenal ulceration	↓	[56]
Gastritis score	↓	[56]

## References

[1] Höcker M., Hohenberger P. (2003). *Helicobacter pylori* virulence factors-one part of a big picture. *The Lancet*.

[2] Amedei A., Della Bella C., Silvestri E., Prisco D., D'Elios M. M. (2012). T cells in gastric cancer: friends or foes. *Clinical and Developmental Immunology*.

[3] Cellini L. (2014). *Helicobacter pylori*: a chameleon-like approach to life. *World Journal of Gastroenterology*.

[4] Algood H. M., Cover T. L. (2006). *Helicobacter pylori* persistence: an overview of interactions between *H. pylori* and host immune defenses. *Clinical Microbiology Reviews*.

[5] Hitzler I., Kohler E., Engler D. B., Yazgan A. S., Müller A. (2012). The role of Th cell subsets in the control of helicobacter infections and in T cell-driven gastric immunopathology. *Frontiers in Immunology*.

[6] Harrington L. E., Hatton R. D., Mangan P. R. (2005). Interleukin 17-producing CD4^+^ effector T cells develop via a lineage distinct from the T helper type 1 and 2 lineages. *Nature Immunology*.

[7] Sakaguchi S., Miyara M., Costantino C. M., Hafler D. A. (2010). FOXP3^+^ regulatory T cells in the human immune system. *Nature Reviews Immunology*.

[8] Zhang C., Yamada N., Wu Y.-L., Wen M., Matsuhisa T., Matsukura N. (2005). Comparison of *Helicobacter pylori* infection and gastric mucosal histological features of gastric ulcer patients with chronic gastritis patients. *World Journal of Gastroenterology*.

[9] Wilson K. T., Crabtree J. E. (2007). Immunology of *Helicobacter pylori*: insights into the failure of the immune response and perspectives on vaccine studies. *Gastroenterology*.

[10] Meyer F., Wilson K. T., James S. P. (2000). Modulation of innate cytokine responses by products of *Helicobacter pylori*. *Infection and Immunity*.

[11] Berenson L. S., Ota N., Murphy K. M. (2004). Issues in T-helper 1 development—resolved and unresolved. *Immunological Reviews*.

[12] Eaton K. A., Benson L. H., Haeger J., Gray B. M. (2006). Role of transcription factor T-bet expression by CD4^+^ cells in gastritis due to *Helicobacter pylori* in mice. *Infection and Immunity*.

[13] Mohammadi M., Nedrud J., Redline R., Lycke N., Czinn S. J. (1997). Murine CD4 T-cell response to *Helicobacter* infection: TH1 cells enhance gastritis and TH2 cells reduce bacterial load. *Gastroenterology*.

[14] Saldinger P. F., Porta N., Launois P. (1998). Immunization of BALB/c mice with *Helicobacter urease* B induces a T helper 2 response absent in Helicobacter infection. *Gastroenterology*.

[15] Garhart C. A., Nedrud J. G., Heinzel F. P., Sigmund N. E., Czinn S. J. (2003). Vaccine-induced protection against *Helicobacter pylori* in mice lacking both antibodies and interleukin-4. *Infection and Immunity*.

[16] Gray B. M., Fontaine C. A., Poe S. A., Eaton K. A. (2013). Complex T cell interactions contribute to *Helicobacter pylori* gastritis in mice. *Infection and Immunity*.

[17] Luzza F., Parrello T., Monteleone G. (2000). Up-regulation of IL-17 is associated with bioactive IL-8 expression in *Helicobacter pylori*-infected human gastric mucosa. *The Journal of Immunology*.

[18] Lundgren A., Strömberg E., Sjöling Å. (2005). Mucosal *FOXP3*-expressing CD4^+^CD25^high^ regulatory T cells in *Helicobacter pylori*-infected patients. *Infection and Immunity*.

[19] Raghavan S., Quiding-Järbrink M. (2012). Immune modulation by regulatory T cells in *Helicobacter pylori*-associated diseases. *Endocrine, Metabolic and Immune Disorders—Drug Targets*.

[20] García M., Bellosillo B., Sánchez-González B. (2012). Study of regulatory T-cells in patients with gastric malt lymphoma: influence on treatment response and outcome. *PLoS ONE*.

[21] McGuirk P., McCann C., Mills K. H. (2002). Pathogen-specific T regulatory 1 cells induced in the respiratory tract by a bacterial molecule that stimulates interleukin 10 production by dendritic cells: a novel strategy for evasion of protective T helper type 1 responses by *Bordetella pertussis*. *The Journal of Experimental Medicine*.

[22] Belkaid Y., Piccirillo C. A., Mendez S., Shevach E. M., Sacks D. L. (2002). CD4^+^CD25^+^ regulatory T cells control *Leishmania* major persistence and immunity. *Nature*.

[23] Raghavan S., Fredriksson M., Svennerholm A. M., Holmgren J., Suri-Payer E. (2003). Absence of CD4^+^Cd25^+^ regulatory T cells is associated with a loss of regulation leading to increased pathology in *Helicobacter pylori*-infected mice. *Clinical & Experimental Immunology*.

[24] Kaparakis M., Laurie K. L., Wijburg O. (2006). CD4^+^ CD25^+^ regulatory T cells modulate the T-cell and antibody responses in *Helicobacter*-Infected BALB/c mice. *Infection and Immunity*.

[25] Mohammadi M., Czinn S., Redline R., Nedrud J. (1996). Helicobacter-specific cell-mediated immune responses display a predominant Th1 phenotype and promote a delayed-type hypersensitivity response in the stomachs of mice. *The Journal of Immunology*.

[26] D'Elios M. M., Manghetti M., de Carli M. (1997). T helper 1 effector cells specific for *Helicobacter pylori* in the gastric antrum of patients with peptic ulcer disease. *Journal of Immunology*.

[27] Eaton K. A., Mefford M., Thevenot T. (2001). The role of T cell subsets and cytokines in the pathogenesis of *Helicobacter pylori* gastritis in mice. *Journal of Immunology*.

[28] Gebert B., Fischer W., Weiss E., Hoffmann R., Haas R. (2003). *Helicobacter pylori* vacuolating cytotoxin inhibits T lymphocyte activation. *Science*.

[29] Sundrud M. S., Torres V. J., Unutmaz D., Cover T. L. (2004). Inhibition of primary human T cell proliferation by *Helicobacter pylori* vacuolating toxin (VacA) is independent of VacA effects on IL-2 secretion. *Proceedings of the National Academy of Sciences of the United States of America*.

[30] Gerhard M., Schmees C., Voland P. (2005). A secreted low-molecular-weight protein from *Helicobacter pylori* induces cell-cycle arrest of T cells. *Gastroenterology*.

[31] Schmees C., Prinz C., Treptau T. (2007). Inhibition of T-cell proliferation by *Helicobacter pyloriγ*-glutamyl transpeptidase. *Gastroenterology*.

[32] Zabaleta J., McGee D. J., Zea A. H. (2004). *Helicobacter pylori* arginase inhibits T cell proliferation and reduces the expression of the TCR zeta-chain (CD3zeta). *Journal of Immunology*.

[33] Wang J., Brooks E. G., Bamford K. B., Denning T. L., Pappo J., Ernst P. B. (2001). Negative selection of T cells by *Helicobacter pylori* as a model for bacterial strain selection by immune evasion. *Journal of Immunology*.

[34] Meyer F., Ramanujam K. S., Gobert A. P., James S. P., Wilson K. T. (2003). Cutting edge: cyclooxygenase-2 activation suppresses Th1 polarization in response to *Helicobacter pylori*. *The Journal of Immunology*.

[35] Pellicanò A., Imeneo M., Leone I., Larussa T., Luzza F. (2007). Enhanced activation of cyclooxygenase-2 downregulates Th1 signaling pathway in *Helicobacter pylori*-infected human gastric mucosa. *Helicobacter*.

[36] Larussa T., Leone I., Suraci E. (2015). Enhanced expression of indoleamine 2,3-dioxygenase in *Helicobacter pylori*-infected human gastric mucosa modulates Th1/Th2 pathway and interleukin 17 production. *Helicobacter*.

[37] Bimczok D., Grams J. M., Stahl R. D., Waites K. B., Smythies L. E., Smith P. D. (2011). Stromal regulation of human gastric dendritic cells restricts the Th1 response to *Helicobacter pylori*. *Gastroenterology*.

[38] Bergman M. P., Engering A., Smits H. H. (2004). *Helicobacter pylori* modulates the T helper cell 1/T helper cell 2 balance through phase-variable interaction between lipopolysaccharide and DC-SIGN. *The Journal of Experimental Medicine*.

[39] Toller I. M., Hitzler I., Sayi A., Mueller A. (2010). Prostaglandin E2 prevents *Helicobacter*-induced gastric preneoplasia and facilitates persistent infection in a mouse model. *Gastroenterology*.

[40] Ouyang W., Kolls J. K., Zheng Y. (2008). The biological functions of T helper 17 cell effector cytokines in inflammation. *Immunity*.

[41] Das S., Suarez G., Beswick E. J., Sierra J. C., Graham D. Y., Reyes V. E. (2006). Expression of B7-H1 on gastric epithelial cells: Its potential role in regulating T cells during *Helicobacter pylori* infection. *Journal of Immunology*.

[42] Lina T. T., Pinchuk I. V., House J. (2013). CagA-dependent downregulation of B7-H2 expression on gastric mucosa and inhibition of Th17 responses during *Helicobacter pylori* infection. *Journal of Immunology*.

[43] Kao J. Y., Zhang M., Miller M. J. (2010). *Helicobacter pylori* immune escape is mediated by dendritic cell-induced Treg skewing and Th17 suppression in mice. *Gastroenterology*.

[44] Oertli M., Sundquist M., Hitzler I. (2012). DC-derived IL-18 drives Treg differentiation, murine *Helicobacter pylori*-specific immune tolerance, and asthma protection. *The Journal of Clinical Investigation*.

[45] Oertli M., Müller A. (2012). *Helicobacter pylori* targets dendritic cells to induce immune tolerance, promote persistence and confer protection against allergic asthma. *Gut Microbes*.

[46] Maloy K. J., Salaun L., Cahill R., Dougan G., Saunders N. J., Powrie F. (2003). CD4^+^CD25^+^ TR cells suppress innate immune pathology through cytokine-dependent mechanisms. *Journal of Experimental Medicine*.

[47] Mitchell P. J., Afzali B., Fazekasova H. (2012). *Helicobacter pylori* induces in-vivo expansion of human regulatory T cells through stimulating interleukin-1*β* production by dendritic cells. *Clinical & Experimental Immunology*.

[48] Rowland M., Daly L., Vaughan M., Higgins A., Bourke B., Drumm B. (2006). Age-specific incidence of *Helicobacter pylori*. *Gastroenterology*.

[49] Luzza F., Suraci E., Larussa T., Leone I., Imeneo M. (2014). High exposure, spontaneous clearance, and low incidence of active *Helicobacter pylori* infection: the Sorbo San Basile study. *Helicobacter*.

[50] Serrano C., Wright S. W., Bimczok D. (2013). Downregulated Th17 responses are associated with reduced gastritis in *Helicobacter pylori*-infected children. *Mucosal Immunology*.

[51] Luzza F., Parrello T., Sebkova L. (2001). Expression of proinflammatory and Th1 but not Th2 cytokines is enhanced in gastric mucosa of *Helicobacter pylori* infected children. *Digestive and Liver Disease*.

[52] Lopes A. I., Quiding-Jarbrink M., Palha A. (2005). Cytokine expression in pediatric *Helicobacter pylori* infection. *Clinical and Diagnostic Laboratory Immunology*.

[53] Marchant A., Goldman M. (2005). T cell-mediated immune responses in human newborns: ready to learn?. *Clinical and Experimental Immunology*.

[54] Freire de Melo F., Rocha G. A., Rocha A. M. (2014). Th1 immune response to *H. pylori* infection varies according to the age of the patients and influences the gastric inflammatory patterns. *International Journal of Medical Microbiology*.

[55] Bhuiyan T. R., Islam M. M., Uddin T. (2014). Th1 and Th17 responses to *Helicobacter pylori* in Bangladeshi infants, children and adults. *PLoS ONE*.

[56] Bontems P., Aksoy E., Burette A. (2014). NF-*κ*B activation and severity of gastritis in *Helicobacter pylori*-infected children and adults. *Helicobacter*.

[57] Arnold I. C., Lee J. Y., Amieva M. R. (2011). Tolerance rather than immunity protects from *Helicobacter pylori*-induced gastric preneoplasia. *Gastroenterology*.

[58] Cho K. Y., Cho M. S., Seo J. W. (2012). FOXP3^+^ regulatory T cells in children with *Helicobacter pylori* Infection. *Pediatric and Developmental Pathology*.

[59] Harris P. R., Wright S. W., Serrano C. (2008). *Helicobacter pylori* gastritis in children is associated with a regulatory T-cell response. *Gastroenterology*.

[60] Freire de Melo F., Rocha A. M., Rocha G. A. (2012). A regulatory instead of an IL-17 T response predominates in *Helicobacter pylori*-associated gastritis in children. *Microbes and Infection*.

[61] Rad R., Brenner L., Bauer S. (2006). CD25+/Foxp3+ T cells regulate gastric inflammation and Helicobacter pylori colonization in vivo. *Gastroenterology*.

[62] Chen W., Shu D., Chadwick V. S. (2001). *Helicobacter pylori* infection: mechanism of colonization and functional dyspepsia reduced colonization of gastric mucosa by *Helicobacter pylori* in mice deficient in interleukin-10. *Journal of Gastroenterology and Hepatology*.

[63] Karnes W. E., Samloff I. M., Siurala M. (1991). Positive serum antibody and negative tissue staining for *Helicobacter pylori* in subjects with atrophic body gastritis. *Gastroenterology*.

[64] Zawahir S., Czinn S. J., Nedrud J. G., Blanchard T. G. (2013). Vaccinating against *Helicobacter pylori* in the developing world. *Gut Microbes*.

[65] Akhiani A. A., Pappo J., Kabok Z. (2002). Protection against *Helicobacter pylori* infection following immunization is IL-12-dependent and mediated by Th1 cells. *The Journal of Immunology*.

[66] Garhart C. A., Heinzel F. P., Czinn S. J., Nedrud J. G. (2003). Vaccine-induced reduction of *Helicobacter pylori* colonization in mice is interleukin-12 dependent but gamma interferon and inducible nitric oxide synthase independent. *Infection and Immunity*.

